# Comparison of macronutrient content in human milk measured by mid-infrared human milk analyzer and reference methods

**DOI:** 10.1038/s41372-018-0291-8

**Published:** 2018-12-14

**Authors:** Francesca Giuffrida, Sean Austin, Denis Cuany, Belén Sanchez-Bridge, Karin Longet, Emmanuelle Bertschy, Julien Sauser, Sagar K. Thakkar, Le Ye Lee, Michael Affolter

**Affiliations:** 1 Nestlé Research, Vers-chez-les Blanc, P.O. Box 44, 1000 Lausanne 26, Switzerland; 2 The Children’s Medical Institute, National University Hospital, Singapore, Singapore; 3 Department of Pediatrics, Yong Loo Lin School of Medicine, National University of Singapore, Singapore, Singapore

**Keywords:** Paediatrics, Health services

## Abstract

**Objective:**

The study aims at evaluating mid-infrared human milk analyzer (HMA) accuracy and precision, in human milk (HM).

**Study design:**

Röse-Gottlieb, high-performance anion exchange chromatography-pulsed amperometric detection (HPAEC-PAD), Kjeldahl and amino acid analysis (AA) were selected as references for total fat, lactose and total protein determination.

**Results:**

No significant difference was observed in lactose content between HMA and HPAEC-PAD. Significant differences were observed in fat and protein content between HMA and reference methods. However, the difference in fat content was lower than 12%, and therefore within the variability declared by supplier. For protein determination, the BCA protein assay was selected. No significant differences were observed in total protein content measured by BCA assay, Kjeldahl and AA methods.

**Conclusions:**

HMA was reliable for the quantification of total fat and lactose content, but not for total protein one. The latter was measured by BCA assay, which yielded comparable results to Kjeldahl and AA methods.

## Introduction

Human milk (HM) is recognized as the normative standard of nutrition for infants for the first 6 months of life (http://pediatrics.aappublications.org/content/early/2012/02/22/peds.2011-3552). The absence of breastfeeding has been associated with increased risk of developing childhood disorders such as atopic eczema [1], obesity and/or overweight [2]. HM nutritive and non-nutritive bioactive composition varies depending on length of gestation, stage of lactation and time of day; nutrient composition also varies within a feed and changes according to dietary intakes [3].

The major components of human milk are macronutrients, *i.e.*, lipids, carbohydrates and proteins. These macronutrients are most abundant and second only to water content in maternal milk. Conventional analyses of these macronutrients have required individual and dedicated methodologies with need of relative large volumes of milk. These methods are also time and labor intensive with longer turn-around time for analyses. However, they do have the advantage of specificity, accuracy and repeatability. Typically, these methods have been used for research purposes rather than clinical application of personalized and fortified nutrition for preterm infants in neonatal intensive care units (NICUs). In a NICU, preterm infants are usually fed expressed mothers’ own milk that may be fortified with single or multiple nutrient fortifiers based on the native macronutrient content. However, this approach is imperative on rapid measurement of macronutrient content in large set of mothers’ own milk with relatively low amount of HM sample. For this purpose, human milk analyzers (HMAs) based on mid-infrared transmission spectroscopy have been developed. On such example is MIRIS HMA which consists of an emitter, cuvette and detector. The HMA is calibrated against established standard reference methods routinely used in the dairy industries: ISO (International Organization for Standardization) and IDF (International Dairy Federation) recommend Röse-Gottlieb [4] for fat and Kjeldahl [5] for total protein or true protein (true protein measurement equals crude protein minus non-protein nitrogen). The reference value for carbohydrate content is calculated from the difference between total solids minus fat, protein and ash. Total solids are measured by oven-drying.

Several studies that compare methodologies and validate commercially available HMA instruments have been published [6–12]. However, the results are variable and conflicting with regard to the performance, accuracy and reproducibility of the MIR-based measurements.

The aim of this study was to compare HMA and reference methods, i.e., Röse-Gottlieb extraction [4], Kjeldahl [5] and high-performance anion exchange chromatography with pulsed amperometric detection (HPAEC-PAD) [13], for fat, protein and lactose measurement, respectively, and utilize the HMA system to quantify those macronutrients in HM samples.

## Materials and methods

### Human milk samples

Six samples of fully expressed HM were acquired from Lee Biosolutions (Maryland Heights, MO, USA; www.leebio.com). Additionally, HM samples (*n* = 150) at different lactation stages were collected in the framework of a clinical study registered with ClinicalTrial.gov with the handler NCT01805011. The protocol and collection of HM was reviewed and approved by the local ethical committee of Singapore and informed consent was obtained from all subjects. The study took place at the National University of Singapore. Volunteer mothers of term infants, who were apparently healthy and non-smokers (*n* = 50; 31.1 ± 3.1 years old) provided breast milk samples (about 30 mL; 30, 60 and 120 days postpartum). Samples were collected after full expression from one breast using a hospital grade electrical breast pump and while the baby was fed on the other breast to produce a satisfactory letdown in the absence of suckling response. All efforts were made to collect HM that included fore-milk, mid-milk and hind-milk as a representation of one feed and to avoid within feed variation of lipid and other nutrient contents. Approximately 30 mL of milk was split into two conical polypropylene tubes (15 mL) for this study and the rest was fed to the infant. Samples collected for research were stored at −80 °C and shipped on dry ice for analyses to Nestlé Research, Vers-chez-les Blanc, Switzerland.

### Röse-Gottlieb analyses

Six pools of fully expressed HM were analyzed for their content of total fat (*n* = 12) by a modified Röse-Gottlieb method where the sample size was decreased to 1 g and solvent volumes reduced in order to maintain the same proportions of sample to solvent ratio as in the reference method.

### HPAEC-PAD analyses

Six pools of fully expressed HM were analyzed for their content in total lactose (*n* = 12) by HPAEC-PAD. Samples were diluted with hot water (70 °C, 25–30 min). After cooling, the solution was diluted to 100 mL in a volumetric flask (further dilutions were made if necessary to keep the sample concentration within the range of the standard curve). Aliquots of the samples were briefly spun in a centrifuge (12,000 × *g*) to remove particles prior to injecting on the HPAEC-PAD system. Sugars present in the sample were separated on a base-stable polymeric column (CarboPac PA20, 3 × 150 mm, 6.5 µm, Thermo Scientific Dionex) using eluents: A sodium hydroxide (NaOH) solution (300 mmol L^−1^), B water and C sodium acetate (NaOAc) (500 mmol L^−1^) containing NaOH (150 mmol L^−1^). The gradient is shown in Table 1. Lactose was detected by pulsed amperometry. Post-column addition of NaOH (300 mmol L^−1^, 0.2 mL min^−1^) was added prior to the PAD to optimize baseline stability, detector sensitivity and linear range. Quantification was performed using an external calibration curve.

**Table 1:** Elution program for determination of lactose

Time (min)	Flow rate (mL min^−1^)	Eluent ‘A’ (%)	Eluent ‘B’ (%)	Eluent ‘C’ (%)
0.0	0.5	2.0	98.0	0.0
1.0	0.5	2.0	98.0	0.0
12.0	0.5	5.0	95.0	0.0
21.0	0.5	22.4	65.6	12.0
21.1	0.5	0.0	0.0	100.0
26.0	0.5	0.0	0.0	100.0
26.1	0.5	100.0	0.0	0.0
31.0	0.5	100.0	0.0	0.0
31.1	0.5	2.0	98.0	0.0
37.0	0.5	2.0	98.0	0.0

### Kjeldahl analyses

Six different pools of full expressed HM were analyzed for their content in total proteins (*n* = 12) using the Kjeldahl method (using a conversion factor of 6.25). Analyses were outsourced to external laboratory certified to ISO17025 (Neotron SPA, Modena, Italy).

### BCA analyses

The total protein content of the HM samples was determined using the bicinchoninic acid (BCA) Protein Assay kit (Pierce^®^ BCA protein Assay Kit, http://www.piercenet.com/instructions/2161296.pdf, Thermo Fisher) according to the instructions provided with the kit.

### Amino acid analyses

Six different pools of fully expressed HM were also analyzed for their content in total proteins (*n* = 6) using the amino acid (AA) method based on high-performance liquid chromatography with ultraviolet and fluorimetric detectors (total AA content after acid hydrolysis including Met and Cys, as well as Trp after alkaline hydrolysis). Analyses were outsourced to an external laboratory certified to ISO17025 (Ansynth Service B.V., Roseendaal, The Netherlands).

### Analysis of human milk by MIRIS human milk analyzer

Fat, protein and lactose contents were determined in HM samples. The device employed for analyses was a HMA (MIRIS AB, Uppsala, Sweden) using the XMA-SW software version 2.87. This HMA is based on semi-solid mid-infrared (MIR) transmission spectroscopy. The wavebands used are specific for the functional carbonyl groups (5.7 µm) for fat determination; amide groups (6.5 µm) for protein determination; and hydroxyl groups (9.6 µm) for carbohydrate determination. Prior to analysis, a daily calibration check was performed using the calibration solution provided by the supplier. All samples were homogenized for 3 × 10 s using the MIRIS sonicator (MIRIS AB, Uppsala, Sweden) as recommended by MIRIS and were kept in a water bath at 40 °C prior to measurement. Homogenized samples (1 mL) were injected into the flow cell and measured within a minute. Once the analysis was completed, the built-in cell and all lines were rinsed with deionized water. After five milk samples, the system was cleaned with the recommended MIRIS cleaning solution. An in-house control sample as well as a calibration standard provided by the manufacturer were analyzed after every tenth measurement for quality control purposes.

### Statistical analyses

The Aspin-Welsh test was performed to compare average or median and standard deviation values.

## Results

### Total fat determination

The Röse-Gottlieb method was used as the reference method for the quantification of total fat. In order to decrease the sample size needed for analysis, the method was slightly modified: sample size was decreased to 100 μL and solvent volumes reduced in order to maintain the same proportions sample/solvent of the reference method. In order to evaluate the trueness of the HMA results for the quantification of total fat, different aliquots of pooled HM samples were analyzed six times in duplicate by the HMA (*n* = 12) and the modified Röse-Gottlieb method (*n* = 12) (Table S1 Supplementary Material).

The amount of fat determined with HMA (3.34 ± 0.22 g 100 mL^−1^) was significantly different (*p* < 0.05) from the amount of fat determined with the modified Röse-Gottlieb method (3.63 ± 0.19 g 100 mL^−1^). However, the difference in total fat content between HMA and reference method was lower than 12%, and therefore within the measurement variability announced by the supplier. Nevertheless, when the same HM pool was analyzed (*n* = 12) with two different MIRIS HMA instruments, variability up to 18% was obtained with average values at 2.8 ± 0.1 and 2.3 ± 0.1 g 100 mL^−1^.

Both techniques showed acceptable repeatability (CV(r)), 2.5 and 6.8%, and intermediate reproducibility values (CV(iR)), 5.3 and 14.7%, respectively.

**Fig. 1:**
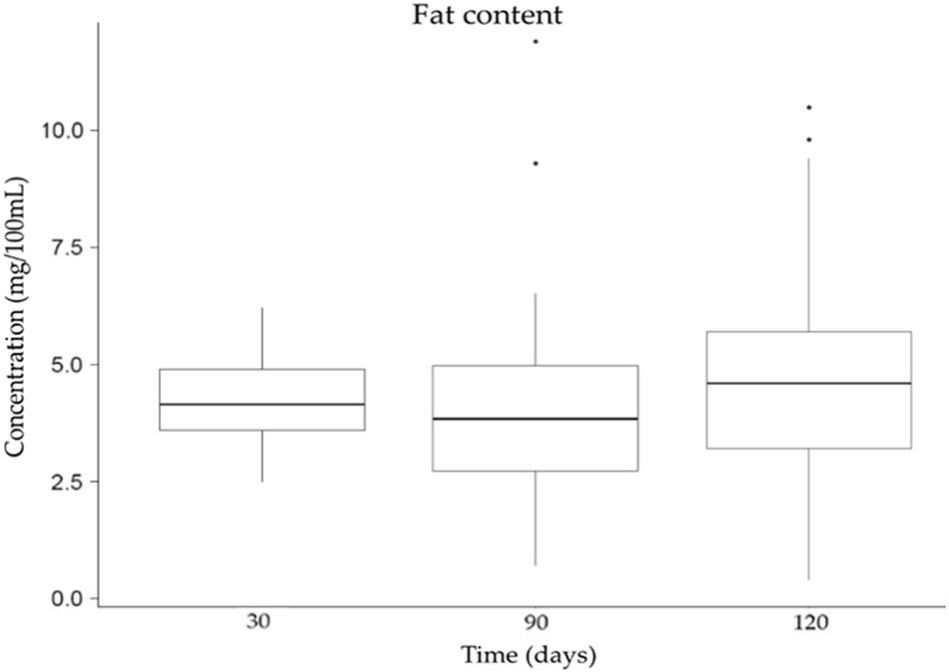
Total fat content in fully expressed HM samples collected at 30, 60 and 120 days postpartum, analyzed by MIRIS HMA. Error bars represent standard deviation

HM samples (*n* = 150) collected at 30, 60 and 120 days postpartum were analyzed with the HMA for their total fat content (Fig. 1). The average lipid content in HM varied from 4.17 ± 0.92 g 100 mL^−1^ (at 30 days) to 4.65 ± 2.10 g 100 mL^−1^ (at 120 days), respectively [14].

These samples were also analyzed for their content in total fatty acids [14] and correlation between total fatty acids measured by gas chromatography and total fat determined by HMA evaluated. A positive correlation (*R*^2^ = 0.942) between total fatty acids and total fat contents was observed. However, a systematic bias was identified, and therefore the methodologies were not comparable with regard to the trueness.

### Lactose determination

The HPAEC-PAD method was selected as the reference method for the quantification of lactose. In order to evaluate the trueness of the HMA measurement for the quantification of lactose, different aliquots of pooled HM samples were analyzed six times in duplicate by the HMA (*n* = 12) and the HPAEC-PAD method (*n* = 12) (Table S2 Supplementary Material).

The amount of lactose determined with the HMA (6.58 g 100 mL^−1^) was not significantly different (*p* > 0.05) from the amount of lactose measured by HPAEC-PAD (6.54 g 100 mL^−1^) (Table 2S). Both techniques showed acceptable CV(r), 3.8 and 0.9%, and CV(iR) values, 5.6 and 3.8%, respectively.

**Fig. 2:**
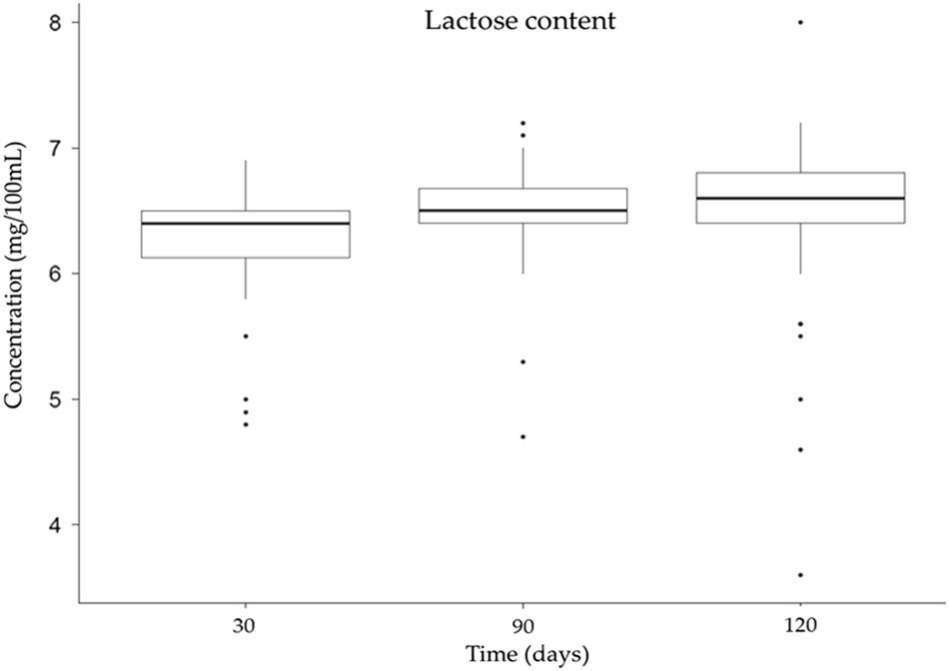
Total lactose content in fully expressed HM samples collected at 30, 60 and 120 days postpartum, analyzed by MIRIS HMA. Error bars represent standard deviation

HM samples (*n* = 150) collected at 30, 60 and 120 days postpartum, were analyzed with the HMA for their total lactose content (Fig. 2). The average lactose content in HM varied from 6.28 ± 0.45 g 100 mL^−1^ (at 30 days) to 6.44 ± 0.69 g 100 mL^−1^ (at 120 days) [14].

### Protein determination

The HMA instrument provides total (crude) and true protein values, whereas the Kjeldahl method, based on a complete combustion of the sample, measures total nitrogen and is considered as reference method for the determination of total protein (conversion factor used 6.25). The amino acid composition, based on chromatography separation of total amino acids liberated after hydrolysis, was also used for the determination of total protein (conversion factor used 100/116).

#### Total protein content

In order to evaluate the accuracy of the HMA for the quantification of total protein content, different aliquots of pooled HM samples were analyzed 12 times in duplicate by the HMA (*n* = 24) and 6 times in duplicate by the Kjeldahl and BCA method (*n* = 12) (Table S3 Supplementary Material).

The total protein content determined with the HMA (0.68 ± 0.09 g 100 mL^−1^) was significantly different (*p* < 0.05) from the amount of total protein content determined by Kjeldahl method (1.16 ± 0.01 g 100 mL^−1^) (Table 3S). The difference in total protein content between HMA and reference method was higher than 15%.

In order to identify alternative methods for rapid and robust total protein determination requiring low volumes of sample, several colorimetric protein assays were pre-screened (Lowry, Bradford, BCA, no data shown) and the BCA protein assay (Pierce® BCA protein Assay Kit) was selected based on its simplicity, sensitivity and robustness with raw milk samples for further evaluation and validation. The assay combines the reduction of Cu^2+^ to Cu^+^ by the protein in an alkaline medium with colorimetric detection of the cuprous cation Cu2+ using a reagent containing BCA. The purple-colored reaction product is formed by the chelation of two molecules of BCA with one cuprous ion. This water-soluble complex exhibits a strong absorbance at 562 nm that is nearly linear over a broad concentration range (20–2000 μg mL^−1^).

The total protein content determined with the BCA assay (1.14 ± 0.13 g 100 mL^−1^) was not significantly different (*p* > 0.05) from the protein content determined by Kjeldahl method (1.16 ± 0.01 g 100 mL^−1^) (Table 3S).

The precision of the BCA Protein Assay kit was evaluated for total protein determination by calculating the repeatability and the intermediate reproducibility (Table S4 Supplementary Material). These results showed CV(r) < 11% and CV(iR) < 15%.

Results from the AA analysis of the six different HM sample pools were also compared to the Kjeldahl and BCA data and no significant difference (*p* > 0.05) was observed (Table S5 Supplementary Material).

#### Total protein content in human milk

**Fig. 3:**
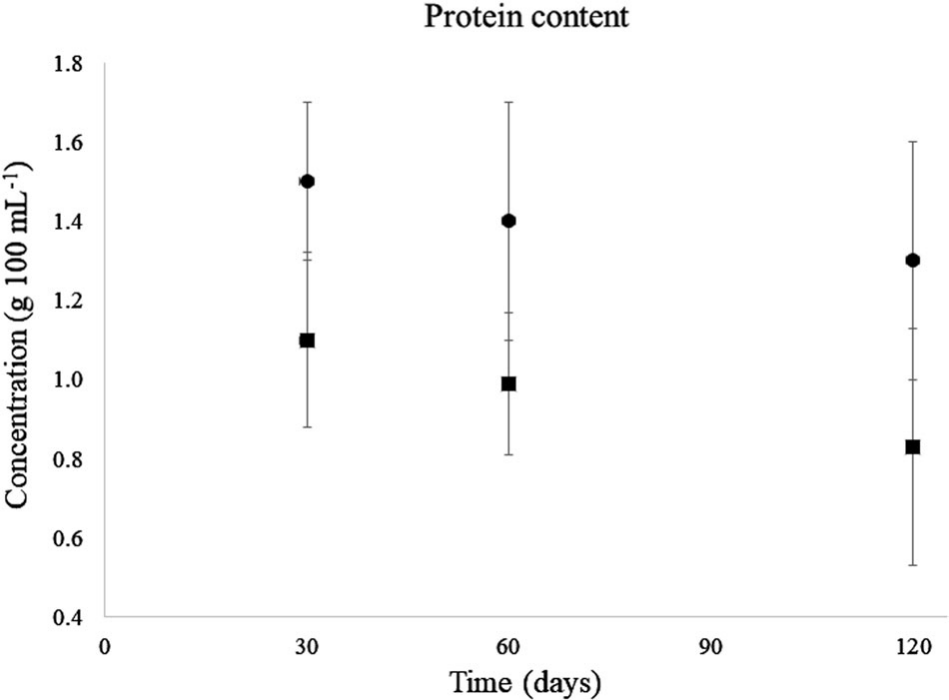
Total protein content in fully expressed HM samples collected at 30, 60 and 120 days postpartum, analyzed by MIRIS HMA and BCA assay. Circles represent BCA results and squares HMA results. Error bars represent standard deviation

HM samples (*n* = 150) were analyzed with the HMA and BCA kit for their total protein content (Fig. 3) The average protein content in HM measured with the HMA varied from 1.10 ± 0.22 g 100 mL^−1^ (at 30 days) to 0.83 ± 0.30 g 100 mL^−1^ (at 120 days) and from 1.5 to 1.3 g 100 mL^−1^ with the BCA assay.

## Discussion

The objective of this work was to assess the accuracy of the HMA instrument compared to reference methods for the analysis of macronutrients in large set of HM samples. Röse-Gottlieb, HPAEC-PAD, Kjeldahl and AA methods were selected as reference methods for the determination of total fat, lactose, total protein and true protein in HM, respectively.

Reference methods are considered the gold standards, although they may also entail some disadvantages such as sample volume required for analyses, low throughput and equipment cost/availability. The Kjeldahl method is considered the reference method for protein analysis but the HM sample volume required is 5 mL versus 1 mL for the HMA and 100 μL for BCA analyses. The Röse-Gottlieb method is considered as the reference method for analysis of fat, but it is only possible to analyze 12 samples a day versus over 60 samples that can be analyzed by the HMA. Finally, the reference method for analysis of lactose, HPAEC-PAD chromatography, requires specific equipment and trained operators versus minimal training requirement for the MIRIS HMA. Considering these factors, the HMA promises a higher throughput, relatively less volume of HM usage, and to be executed by a user who has undergone relatively simple training and needs a simpler know-how.

No significant differences were found between the HMA and reference method for the determination of lactose content in HM, whereas significant differences were found for the determination of fat content. However, the difference in total fat content between HMA and the reference method was lower than 12%, which was within the variability reported by the HMA supplier. Total fatty acids were also measured [14] and correlation with HMA evaluated. Despite the positive correlation (*R*^2^ = 0.942) between total fatty acids and total fat contents, a systematic bias was identified, and therefore the methodologies were not comparable with regard to the trueness. The majority of fatty acids are esterified to glycerol as triacylglycerol and about 0.2–2% are found esterified to other molecules such as cholesterol, phospholipids and gangliosides. Therefore, we can estimate that total fatty acids represent about 98% of total fat and this could explain the identified systematic bias.

No agreement was observed between the HMA and reference Kjeldahl method for total protein content, which was underestimated by the HMA instrument. For total protein content, the difference between HMA and reference methods was above 15%, and therefore the HMA was considered not suitable for protein quantification in human milk.

Based on these results, colorimetric assays were evaluated to determine the total protein content in HM. The BCA assay was found to perform accurately and robustly with no significant differences observed when compared against the Kjeldahl and AA methods. Some previous publications compared various colorimetric assays, including the BCA assay, for the determination of the protein content in HM [15, 16]. Lönnerdal et al. [15] showed that the BCA method consistently overestimated Kjeldahl protein by 30%, whereas Keller and Neville [16] claimed that the BCA assay showed the least difference in values and the greatest precision. The protocol used for sample preparation, *i.e.,*dilution, incubation time and temperature, seems to critically influence the accuracy of the BCA method [17], and therefore particular care must be taken to precisely control every upfront step prior to the BCA assay.

**Fig. 4:**
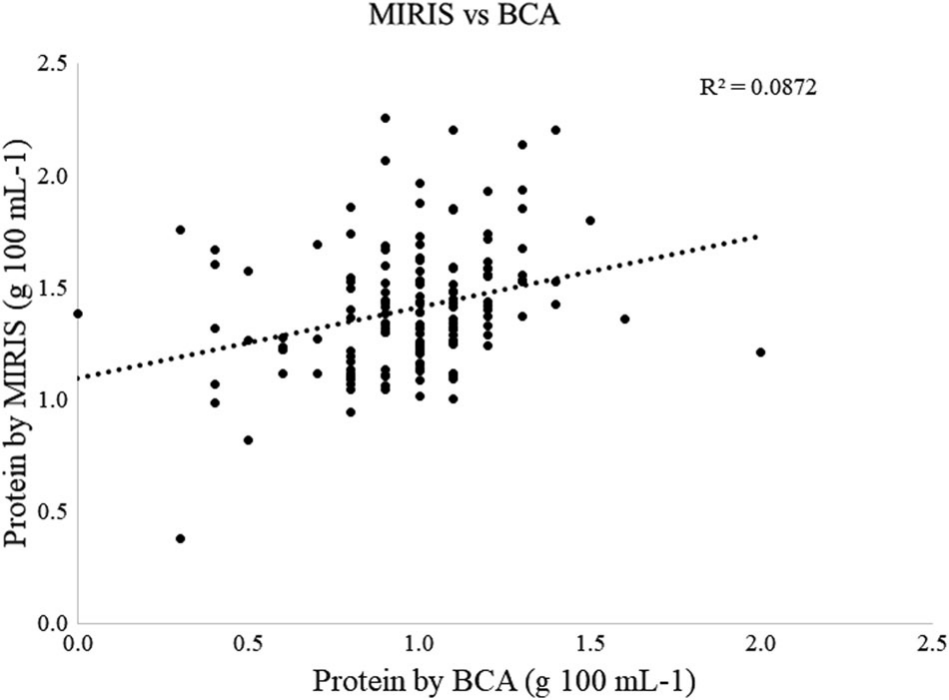
Correlation between total protein content in individual fully expressed HM samples collected at 30, 60 and 120 days postpartum, analyzed by MIRIS HMA and BCA assay

Comparison of the HMA and BCA protein data revealed that the measured concentrations did not follow a linear relation, which could be adjusted by a correction (or bias) factor (Fig. 4). Individual values fluctuated independently for each sample and the adjustment options (slope, bias) provided by the MIRIS instrument software could not correct for the observed differences. The BCA assay was thus selected to determine the total protein content in HM.

Several investigators have evaluated the HMA filter-based MIR analyzer, with variable and conflicting results [6, 7, 9, 11, 12, 18, 19]. Silvestre et al. [18] compared MIRIS HMA versus Gerber, Bradford and chloramine-T methods for fat, proteins and lactose determination, respectively, reporting overestimation of lipid content and underestimation of protein and lactose content. These results are in agreement with our observations only for the protein content. The discrepancies for fat and lactose determination may be explained by the choice of the reference methods. The Gerber method is based on protein precipitation and further separation of fat by centrifugation. A collaborative study performed on crude and homogenized pasteurized cow milk [19] showed that the Gerber method underestimated the amount of fat (0.02–0.06%) compared to the official method Röse-Gottlieb. The chloramine-T method is based on the reducing characteristic of lactose. Chloramine T (an oxidant) is added, and is quantitatively reduced by the lactose. Excess chloramine-T is reacted with acidified potassium iodide (KI), which is oxidized to iodine. The iodine released is determined by titration with sodium thiosulphate, and therefore any agent capable of interfering with the chloramine-T and KI reaction may lead to over- or underestimation of lactose content.

Casadio et al. [20] compared MIRIS HMA versus esterified fatty acids, Bradford and enzymatic spectroscopic methods for fat, proteins and lactose determination, respectively, reporting overestimation of lipid and protein content and underestimation of lactose content. The authors state that their results were not unexpected given that analytical techniques used to quantify the MIRIS HMA calibrants were different to the reference laboratory techniques.

Fush et al. [6] compared MIRIS HMA versus Mojonnier (automated Röse-Gottlieb), Dumas and liquid chromatography–tandem mass spectrometry methods for fat, proteins and lactose determination, respectively, reporting accurate and precise results for lipid and protein content and difficulties in obtaining accurate values for lactose, probably due to the presence of oligosaccharides which interfere with lactose measurement.

Recently, Zhu et al. [11] compared MIRIS HMA to Röse-Gottlieb, micro-Kjeldahl and HPAEC-PAD, reporting accurate and precise values only for lactose; protein content was underestimated and fat content overestimated when analyzed by MIRIS HMA. The reference methods selected by Zhu et al. [11] are similar to ours, and the disagreement between our findings with regard to fat content does not have a reasonable explanation except, as recently shown by Buffin et al. [12], differences in the model and software version of MIRIS HMA could be a factor. We observed that when the same HM pool was analyzed for total fat (*n* = 12) with two different MIRIS HMA instruments, variability up to 18% was obtained with average values at 2.8 ± 0.1 and 2.3 ± 0.1 g 100 mL^−1^. Therefore, the variability observed in total fat content determination among different MIRIS HMA machines requires strict monitoring of the instrument using established reference materials.

Groh-Wargo et al. [9] have investigated a different filter-based middle infrared analyzer (Calais Human Milk Analyzer, North American Instruments, Lake Oswego, OR) and observed a good correlation with reference methods for proteins (Kjeldahl) and fat (Mojonnier), but difficulties in obtaining accurate values for lactose (compared to HPAEC-PAD) which nevertheless remained comparable. Lastly, Smilowitz et al. [7] demonstrated high accuracy in measurements of macronutrients by Fourier transform mid-infrared spectroscopy with an instrument modified to allow analyses of small volume of samples. MIR seems to have potential, but building good calibration models based on appropriate reference methods is critical.

Finally, 150 fully expressed HM samples collected at 30, 60 and 120 days postpartum were analyzed by HMA for the determination of total fat and lactose, and by the BCA assay for determination of total protein. In order to standardize sample collection, HM samples were collected at the same time of the day, from the same breast, and all efforts were made to collect HM that included fore-milk, mid-milk and hind-milk as a representation of one feed and to avoid within-feed variation of lipid and other nutrient contents. Our results for all macronutrients matched the values and trajectories previously reported in literature [21].

## Conclusions

The results of this investigation demonstrate that the MIRIS HMA is suitable for the quantification of total fat and lactose in HM, with regard to trueness and precision. Analyses of HM by filter-based MIR spectroscopy correlate well with the reference methods and offers an easy and accurate method to analyze fat and lactose in HM. However, discrepancy in fat content for the same set of samples was observed between different MIRIS HMA machines.

Despite the suppliers’ recommendation, we found HMA not suitable for the quantification of total protein content in HM. However, since the reference method (Kjeldahl) requires large volumes of human milk for analyses, the BCA Protein Assay kit was evaluated and found suitable with regard to trueness and precision for the quantification of total protein content in human milk.

Since our objective was to check for the performance of HMA on HM fat, protein and lactose quantification, we alert the readers that the instrument accuracy and precision needs to be checked at least once a week for routine analyses. In addition, due to continued changes implemented by the manufacturer, this exercise of accuracy and performance check is warranted on the future hardware versions and software releases.

## Supplementary information

Supplementary material
