# Supplementary material for: Comparison of macronutrient content in human milk measured by mid-infrared human milk analyzer and reference methods
Source: J Perinatol. 2018 Dec 14;39(3):497–503. doi: 10.1038/s41372-018-0291-8 (PMC6514977; doi:10.1038/s41372-018-0291-8)
Supplement: Supplementary file 1 — Supplementary material [file 41372_2018_291_MOESM1_ESM.docx]

Supplementary material contains tables listing accuracy and precision data of the methodologies used to evaluate the performance of MIRIS human milk analyzer.

Table S1. Median, relative standard deviation of repeatability [CV(r)], and relative standard deviation of intermediate reproducibility [CV(iR)] for total fat content in HM. Results are expressed in g 100 mL^-1^ of product. Analyses were performed in duplicate by the same operator over six days (n=12).

|  | Modified Röse-Gottlieb | | | MIRIS HMA | | |
| --- | --- | --- | --- | --- | --- | --- |
|  | Median | CV(r) | CV(iR) | Median | CV(r) | CV(iR) |
| HM | 3.63±0.19 | 5.3 | 14.7 | 3.34±0.22 | 2.5 | 6.8 |

Table S2. Median, relative standard deviation of repeatability [CV(r)], and relative standard deviation of intermediate reproducibility [CV(iR)] for total lactose content in HM. Results are expressed in g 100 mL^-1^ of product. Analyses were performed in duplicate by the same operator over six days (n=13).

|  | HPAEC-PAD | | | MIRIS HMA | | |
| --- | --- | --- | --- | --- | --- | --- |
|  | Median | CV(r) | CV(iR) | Median | CV(r) | CV(iR) |
| HM | 6.58 | 0.9 | 2.5 | 6.54 | 3.8 | 5.6 |

Table S3. Total protein content for HM determined by Kjeldahl, MIRIS HMA and BCA assay.

|  | MIRIS HMA  (n=24) | Kjeldahl  (n=12) | BCA  (n=12) |
| --- | --- | --- | --- |
| HM (g 100 mL^-1^) | 0.68±0.09 | 1.16±0.01 | 1.14±0.13 |

Table S4. Median, relative standard deviation of repeatability [CV(r)], and relative standard deviation of intermediate reproducibility [CV(iR)] for total protein content in human milk by BCA kit. Results are expressed in g 100 mL^-1^ of product. Analyses were performed in duplicate by the same operator over six days (n = 12).

|  | Median | CV(r) | CV(iR) |
| --- | --- | --- | --- |
| HM 1 (n=12) | 1.33 | 2.4 | 4.6 |
| HM 2 (n=12) | 1.16 | 2.7 | 5.7 |
| HM 3 (n=12) | 1.24 | 4.2 | 8.7 |
| HM 4 (n=12) | 1.28 | 8.5 | 8.1 |
| HM 5 (n=12) | 1.18 | 10.6 | 14.2 |
| HM 6 (n=12) | 1.24 | 3.4 | 6.9 |

Table 5S. Total protein content in human milk measured by Kjeldahl, BCA assay and AA method. Results are expressed in g 100 mL^-1^ of product.

|  | HM1 | HM2 | HM3 | HM4 | HM5 | HM6 |
| --- | --- | --- | --- | --- | --- | --- |
| Kjeldahl (n=12) | 1.27 | 1.08 | 1.03 | 0.98 | 1.03 | 1.18 |
| BCA kit (n=12) | 1.31 | 1.15 | 1.16 | 1.17 | 1.00 | 1.25 |
| AA (n=6) | 1.10 | 0.93 | 0.89 | 0.85 | 0.89 | 1.01 |
